# The control of classical swine fever in wild boar

**DOI:** 10.3389/fmicb.2015.01211

**Published:** 2015-11-06

**Authors:** Volker Moennig

**Affiliations:** Department of Infectious Diseases, Institute for Virology, University of Veterinary MedicineHannover, Germany

**Keywords:** classical swine fever, wild boar, oral vaccination, control, wildlife diseases

## Abstract

Classical swine fever (CSF) is a viral disease with severe economic consequences for domestic pigs. Natural hosts for the CSF virus (CSFV) are members of the family *Suidae*, i.e., Eurasian wild boar (*sus scrofa*) are also susceptible. CSF in wild boar poses a serious threat to domestic pigs. CSFV is an enveloped RNA virus belonging to the pestivirus genus of the *Flaviviridae* family. Transmission of the infection is usually by direct contact or by feeding of contaminated meat products. In recent decades CSF has been successfully eradicated from Australia, North America, and the European Union. In areas with dense wild boar populations CSF tends to become endemic whereas it is often self-limiting in small, less dense populations. In recent decades eradication strategies of CSF in wild boar have been improved considerably. The reduction of the number of susceptible animals to a threshold level where the basic reproductive number is *R*_0_ < 1 is the major goal of all control efforts. Depending on the epidemiological situation, hunting measures combined with strict hygiene may be effective in areas with a relatively low density of wild boar. Oral immunization was shown to be highly effective in endemic situations in areas with a high density of wild boar.

## Introduction

Classical swine fever (CSF) is an acute viral infection of pigs that causes major economic losses especially in countries with dense populations of domestic pigs. A series of outbreaks occurred in the European Union (EU) after the introduction of the non-vaccination policy in 1990. The economic losses caused by an outbreak in The Netherlands in 1997 were as high as 2.3 billion US$ and more than 11 million pigs had to be destroyed. Only a fraction of these pigs were actually infected or suspect of being infected. The rest had to be killed for preventive measures or welfare reasons in areas that have been under movement restrictions for prolonged periods. Other European countries were also affected by serious outbreaks between 1990 and 2001, e.g., Belgium, Czech Republic, Germany, Italy, and Spain (Meuwissen et al., 1999). Except for Australia, North America, and the EU the infection is endemic in most other parts of the world where pigs are kept. The CSF virus (CSFV) is an enveloped particle containing a single stranded RNA with positive polarity. Taxonomically, it belongs to the genus Pestivirus in the family *Flaviviridae*. It is readily inactivated by common disinfectants and detergents, however, in moist environments, e.g., ham, salami type sausages, fresh pork and excretions of infected pigs it can survive for weeks or even months (Edwards, 2000; Kaden et al., 2004). Contaminated meat and meat products are dangerous sources for the spread of CSF or the fresh introduction of the infection into CSF-free regions, respectively. Related agents are bovine viral diarrhea virus, border disease virus of sheep and a number of other pestiviruses that have recently been detected in wild ruminants. Ruminant pestiviruses occasionally infect pigs subclinically and cross-reacting antibodies of the resulting immune response may pose some problems for the serological diagnosis of CSF. The Eurasian wild boar (*sus scrofa*) is equally susceptible to infection with CSFV. In areas with domestic pigs and wild boar the infection is frequently transmitted from domestic pigs to wild boar and vice versa. CSFV has no known reservoir or animal vector other than swine. The virus does not infect humans, however, it can be transmitted experimentally to ruminants and rabbits.

## Clinical signs

Domestic pigs as well as wild boar are highly susceptible to CSFV infection. Clinical signs and pathogenesis of the infection have been extensively studied in domestic pigs. Although, there are fewer data available for wild boar it is safe to assume that there are no significant differences between domestic pigs and wild boar (Kaden et al., 2004). There are three different clinical courses: Acute CSF, chronic and late onset CSF. The latter is the result of prenatal infection (Artois et al., 2002; Moennig et al., 2003).

The acute infection lasts less than 4 weeks and animals either recover or die within this period. When infected with CSFV strains that recently circulated in Europe piglets are getting severely ill and up to 90% of them die within 4 weeks post infection. Pyrexia with temperatures higher than 40°C is a characteristic sign in juvenile animals. Early symptoms are lethargy, conjunctivitis, huddling together, respiratory signs, conjunctivitis, constipation followed by diarrhea and anorexia. Central nervous symptoms are frequent, e.g., convulsions, weakness of hind legs, staggering gait, and incoordination of movement. Immunosuppression and severe leukopenia facilitate secondary infections of the gastrointestinal and/or respiratory tract. Skin and internal organs often display petechial to ecchymotic bleedings. Infected animals are shedding virus through all secretions and excretions. With increasing age of the infected pigs, clinical signs become milder, less specific and most adult pigs recover. First virus-neutralizing antibodies are detectable 2 weeks post infection and convalescent animals have a stable lifelong immunity against CSFV, which is predominantly humoral.

In domestic pigs the chronic form of the disease, which is always fatal, plays an important role, since infected animals shed large amounts of virus until their death. Chronic CSF develops in a few juvenile animals, which fail to respond efficiently to the infection. Affected pigs are not able to clear the virus and the disease lasts longer than 4 weeks. Early symptoms resemble those of the acute infection. During the course of the disease clinical signs become weaker and less specific, including chronic enteritis, wasting and undulating fever. Sick animals shed virus for the rest of their lives and they die between 2 and 4 months post infection. Antibodies may be produced but they are often not detectable since they are complexed by circulating virus. Neither field nor experimental data on this form of CSF in wild boar are available (Artois et al., 2002). It is questionable whether chronically sick wild boar have a survival chance in their natural habitat.

In infected pregnant pigs CSFV—like other pestiviruses—is able to cross the placenta and to infect fetuses. Depending on the stage of gestation and viral virulence the infection has different outcomes in pigs. After intrauterine infection during early pregnancy a number of disorders occur, e.g., stillbirths, abortions, and mummified fetuses. Infection of sows around 80–90 days of pregnancy may lead to the birth of persistently viremic piglets, which can survive for up to 11 months. Often these piglets are not readily recognized, because they appear clinically normal at the time of birth. Occasionally congenital tremor is observed. After birth, their condition deteriorates and they usually show poor growth (“runt”) and wasting. This course of CSF is always fatal and it is called “late onset CSF.” In domestic pig populations these viremic piglets are dangerous virus reservoirs because they shed large quantities of virus during their lifetime. In pregnant wild boar sows intrauterine infection under laboratory conditions also yields persistently viremic piglets (Depner et al., 1995), however, these animals apparently do not play an important role for the perpetuation of CSF in wild boar populations since their survival time likely to be short (Kaden et al., 2005b).

## Diagnosis

In domestic pigs first suspicion for an outbreak of CSF is usually raised by the clinical picture, especially when the severe acute form of disease is observed. However, due to the rather unspecific symptoms, a long list of other infectious diseases has to be considered as differential diagnosis, e.g., African swine fever, Erysipelas, porcine reproductive, and respiratory syndrome, purpura hemorrhagica, porcine circovirus 2 infections, and other infections with high fever not responding to antibiotic treatment. In wild boar typical indicators for a disease outbreak may be an unusual number of pigs found dead or the observation of sick animals with atypical behavior.

Any clinical suspicion of CSF in domestic pigs and wild boar must be verified using laboratory diagnostic methods. Depending on the state of the samples collected from dead or freshly shot wild boar and the technical capabilities of the laboratories involved a number of laboratory techniques can be applied. The methods for viral and serological detection of CSFV infections are well established and there are detailed descriptions in the “Manual of Standards” of the OIE (Anonymous, 2015) and in the “Diagnostic Manual” attached to “Decision 2002/106/EC” issued by the EU Commission (Anonymous, 2002; Greiser-Wilke et al., 2007).

The tissues recommended for the detection of virus are tonsil, lymph-nodes, spleen, ileum, and kidney. For serological tests it should be attempted to collect tissue fluids from shot wild boar.

Standard techniques for the isolation of virus are based on the use of susceptible porcine cell cultures. Cells infected by CSFV are not lysed and therefore the infection must be visualized using indirect methods, e.g., fixing cells and staining viral antigen using mono- or polyclonal antibodies conjugated with enzymes or fluorescent dyes. Although, virus isolation is time consuming and not as sensitive as polymerase chain reaction after reverse transcription (RT-PCR) it is used for the establishment of virus collections and it allows further analysis of the isolate, e.g., genotyping or analysis of viral virulence in animal experiments. Although, CSFV presents no hazard for humans, there is the risk that the virus escapes from the laboratory and might infect susceptible pigs. Therefore, any work involving live virus including its propagation should be carried out in BSL-3 (agricultural) facilities.

For quick results direct antigen detection can be carried out using immunofluorescence or peroxidase staining with polyclonal or monoclonal antibody conjugates on fixed cryosections of organ material. However, the sensitivity of this method is limited and a negative result does not rule out CSF in case of a clinical suspicion. The interpretation of test results is not trivial and it requires experienced laboratory personnel.

In recent years RT-PCR has become the method of choice, since it yields quick results and it is highly sensitive. It can be used for individual as well as pooled samples (Depner et al., 2006).

Serological diagnosis of CSF is performed using either virus-neutralization assay or a commercially available enzyme-linked immunosorbent assay (ELSA). The former is still considered to be the gold standard, however, it is slow, labor intensive, and often the fluids retrieved from wild boar carcasses are not suitable for use in tissue culture based tests.

## Vaccines

Several types of CSF vaccines have been developed for use in domestic pigs. The efficacy of old inactivated preparations was poor, while more recently developed modified live vaccines (MLV) are highly efficacious and have an excellent safety record in pigs of all ages, e.g., the GPE—and a number of variants of the lapinized Chinese strain (C-strain) of CSFV (Bognar and Meszaros, 1963). Currently MLVs are being used worldwide for the prophylactic vaccination of domestic pigs. They are suitable tools for limiting the severe economic effects of CSF in countries with endemic infection and, when properly used in systematic control programs, their use often was and still is a first step toward eradication of the infection (Terpstra, 1991). Once countries are free from CSF vaccination is usually prohibited.

For oral immunization of wild boar several variants of the C-strain have been used. The efficacy of the vaccine virus after oral administration was tested in domestic pigs and wild boar piglets. Whereas, after parenteral immunization protection is already achieved 2–3 days post vaccination, oral vaccination yields a slightly delayed protection against challenge with virulent CSFV about 4 days after application of the vaccine virus. Neutralizing antibodies were demonstrable after 10 days (Kaden and Lange, 2001). In addition the C-strain virus was tested in non-target species, e.g., cattle, goat, sheep, foxes, hares, rabbits, and mice. In none of these species a clinical reaction was observed (Chenut et al., 1999; Kaden et al., 2010).

In early experiments with oral CSF vaccines, e.g., in Romania, C-strain virus was injected into hen eggs which were then used for oral immunization. Success with these baits was variable. Outbreaks in the early 1990s in Germany have prompted another attempt to develop a new generation of baits, partly based on the experiences made with baits used for oral immunization of foxes against rabies. These baits consisted of corn flour, fat, and almond flavor. A plastic blister with 2 ml aliquots of 10^6^ protective doses_50_ of C-strain virus “Riems” was incorporated in the baits (Kaden et al., 2000, 2005a). Since the original baits were too large for uptake by young animals experiments were made to reduce bait size and replace the liquid vaccine formulation by freeze-dried virus (Brauer et al., 2006). On the genetic level differentiation of vaccine virus from field virus can be made using real-time RT-PCR for the detection of sequence variations (Beer et al., 2007).

However, the general disadvantage of conventional MLV is that vaccinated animals cannot be distinguished serologically from convalescent pigs. In order to overcome this impediment the live DIVA (Differentiating Infected from Vaccinated Animals) vaccine CP7_E2alf has been developed. It is based on a bovine virus diarrhea virus backbone containing the major envelope protein (E2) of CSFV strain Alfort. CP7_E2alf induces a solid immunity in wild boar after oral immunization and it did not induce any clinical signs in non-target species after oral inoculation, e.g., calves, young goats, lambs, and rabbits. Neither fever nor leukopenia was registered in the inoculated animals and virus could not be isolated from purified white blood cells or from nasal or fecal excretions. In another experiment it was shown that the vaccine was also innocuous for the target species: no clinical signs, transmission, or shedding of the vaccine virus was observed (Tignon et al., 2010; König et al., 2011; Gabriel et al., 2012). This novel vaccine has been licensed by the European Medicines Agency in 2014 and has the potential to replace conventional MLV in oral immunization of wild boar. Other live DIVA vaccines based on porcine adenovirus as vector for the E2 glycoprotein of CSFV have been developed earlier, however, it is not clear whether they were intended to be used in the control of CSF in wild boar (Hammond et al., 2003).

## Epidemiology

Transmission routes of CSFV are comparable in wild boar and domestic pigs: Virus is mainly transmitted by direct contact with infected animals. Indirect spread by infected feces, food, and carcasses also plays a role. Naïve populations usually get infected accidentally by indirect and sometimes direct contact with infected domestic pigs or by wild boar feeding on garbage on landfills or rest areas where contaminated food had been dumped. There are well documented reports that CSFV may spill over directly or indirectly from wild boar to domestic pigs, e.g., in Germany 92 primary outbreaks of the infection in holdings of domestic pigs in the years 1993–1997 occurred in areas with endemic CSF in wild boar. It was proven that 60% of these cases were caused by direct or indirect contact with wild boar (Teuffert et al., 1997; Fritzemeier et al., 2000).

Until the early 1990s, little was known concerning the significance of wild boar in the epidemiology of CSF and unfortunately there are hardly any published records of historic CSF outbreaks in wild boar. Earlier observations indicated that CSF outbreaks in wild boar were self-limiting (Terpstra, 1987), probably due to high virulence of viral strains circulating at that time.

However, in the last 25 years ample evidence has accumulated in Europe that wild boar may become a dangerous virus reservoir, although many of today's CSF outbreaks with strains of moderate virulence currently circulating in the field will still die out spontaneously. This seems to be true for small populations of about 2000 wild boar or less where the infection seems to be cleared within 1 year. In contrast CSFV tends to persist and become endemic for years in larger populations (Rossi et al., 2005). In addition to population size, host animal density plays a role in virus persistence, since the high turnover in a dense population provides a quicker renewal of young susceptible pigs, thereby increasing the chance of the virus to persist in the population. Thus, population size and density are crucial factors for viral survival in wild boar populations (Artois et al., 2002).

The course of a CSF outbreak in wild boar largely depends on the threshold, i.e., the number of susceptible animals in an affected population in a defined area. The threshold criterion for each infectious disease in wildlife is the basic reproductive number (*R*_0_). *R*_0_ > 1 means that there are enough susceptible animals to allow the number of secondary infections caused by the first case exceed one, and as a consequence the infectious disease will perpetuate and ultimately become endemic. Below the threshold it is expected *R*_0_ < 1 (Hone et al., 1992; Lloyd-Smith et al., 2005), i.e., the absolute number of susceptible animals is so low that the infection is likely to come to an end. When an epidemic is caused by highly virulent variants of CSFV this number has been estimated to be 1.4 susceptible pigs per km^2^ (Hone et al., 1992). Since the threshold number is significantly influenced by the mortality rate due to the infection this number is lower in endemic areas with low virulent variants of CSFV. It has been estimated to be one susceptible pig per km^2^ (Anonymous, 1999). Artois et al. (2002) estimated the threshold value to be approximately 200 susceptible pigs in an area of about 220 km^2^. With more than 200 susceptible animals in the area the *R*_0_ allows an infected pig to infect one or more other susceptible pigs, i.e., *R*_0_ > 1. Consequently the main goal of any effort to control CSF in wild boar is to stop transmission of the virus by reducing the number of susceptible individuals in the infected area until the threshold is reached (Artois et al., 2002; Anonymous, 2009). In endemic situations the majority of adult animals have survived infection and they are immune for the rest of their lives thereby reducing the number of susceptible animals, while juvenile pigs without maternal antibodies are susceptible and they serve as reservoir for CSFV.

Long-term observations suggest that during the last decades populations of wild boar have increased in density and that the species has occupied new territories (Acevedo et al., 2007, 2014; Ruiz-Fons et al., 2008). Main reasons for this expansion are changing biological and ecological parameters, e.g., relatively mild winters due to a gradual climate change leading to longer mating and delivery seasons and improvement of the feed basis due to more intense farming and/or more shelter available in rural areas that have been abandoned (Acevedo et al., 2007). In addition the flexibility of the species to colonize a wide range of new habitats, including even urban areas, intentional introduction for hunting purposes and a decrease or absence of predators have significantly contributed to this expansion. Together with the high reproduction rate these factors have led to the current situation where wild boar is the most widespread and also the most abundant wild ungulate species in Europe. In case of CSF outbreaks in these populations there is a high probability that the virus will persist for a longer period as has been observed in several outbreaks in Germany and France during the last decade of the last century (Laddomada, 2000; Pol et al., 2008). Once introduced CSFV spreads according to the social and spatial structure of the affected populations, i.e., virus transmission within a social group and between groups. Within social groups, the virus is transmitted very efficiently and with high frequency by direct and indirect contact, especially between piglets. In contrast contacts between social groups are limited and virus is mainly spread indirectly by excretions and carcasses of infected animals. Direct transmission between groups during the rutting season through male dispersers or after vigorous drive hunting resulting in the disruption of social order may also occur, or when new social groups are being established. The high reproduction rate of wild boar provides a constant supply of young animals serving as reservoir for CSFV.

## Control measures

In the EU, CSF control in domestic pigs is based on a stamping out strategy, i.e., outbreaks in domestic pigs are eradicated by culling of infected and suspected animals. The measures are accompanied by temporary animal movement restrictions. Prophylactic vaccination is banned. However, when there is the danger of uncontrollable spread of CSF the EU Commission may approve emergency vaccination. This applies for domestic pigs as well as wild boar.

For obvious reasons programs for the control and eradication of CSF in wild boar have to be different from those applied in domestic pigs. When the suspicion of a CSF outbreak in a wild boar population is confirmed, hunting should be banned temporarily in order not to disperse infected animals into uninfected areas. When designing a control program it is essential for epidemiologists and wild life managers to have information about structure and density of the affected animal populations. Stakeholder, e.g., veterinary officers, hunters, and farmers should take part in the planning. The program should address the following issues (Laddomada, 2000):

- A clear delineation of the infected risk area and the definition of a surrounding surveillance area
- Description of measures to be applied to detect infection in wild boar
- Nomination of organizations and persons involved in control measures and establishment of a clear chain of command
- Measures to be taken to control the infection (see below)
- Epidemiological investigations
- Virological and serological controls on animals shot or found dead, according to standard statistical methods.
- Destruction of infected carcasses and strict hygienic measures when carcasses are eviscerated
- Rules for the use of inspected wild boar meat from CSFV-negative animals
- Movement restrictions for domestic pigs in the designated areas
- Criteria for lifting control measures

Environmental factors, in particular natural barriers have to be taken into account when infected and surveillance areas are defined, e.g., rivers, high mountains and major freeways. In addition movement of wild boar and population densities should be taken into consideration. The structure of the local wild boar population and its subpopulations has to be recorded. Animal density should be estimated as accurately as possible and epidemiological data on virological and serological prevalence should be taken into account. However, it is difficult to estimate the density of wild boar populations because these animals prefer dense vegetation and they have a nocturnal activity pattern. Therefore, indirect methods rather than direct counts of pigs are used for the estimation of population density and abundance. A relatively simple method which can be applied in emergency situations, e.g., CSF outbreaks, is hunting bag analysis if possible over several years (Acevedo et al., 2007; Bosch et al., 2012). Other more complex methods are pellet counts (Acevedo et al., 2007) and capture—recapture—for instance, in combination with non-invasive genetic sampling (Hebeisen et al., 2008; Ebert et al., 2010).

With these data at hand a decision on control measures should be made. Depending on geographical conditions and the size and structure of the affected wild boar population these measures may be either “minimal intervention” in the case of small and isolated populations, feeding, fencing, hunting, trapping, oral immunization, or a combination of the above when larger and more complex populations in wide open areas are affected.

### Public awareness and education

Measures to control CSF in wild boar are complex and may be long-lasting. For the success of the program it is therefore very important to first launch an awareness campaign addressing all groups affected or involved in control measures, e.g., veterinary authorities, hunters, farmers, and the local public. Especially, hunters are a critical group because their cooperation is essential and because control programs, e.g., hunting ban or depopulation efforts targeting piglets, are not compatible with their goals and hunting traditions. Many of them do not consider CSF a problem, and therefore educational measures should precede or accompany each control program highlighting the impact of CSF on sports hunting and the general economy. Especially, for vaccination campaigns and targeted shooting of piglets awareness and cooperation of hunters is crucial. Compensatory measures such as bonuses for each piglet shot could be considered to stimulate co-operation of hunters.

### Surveillance and monitoring

In most cases samples for surveillance and monitoring are provided by hunters. Quality of samples is often poor since it depends on the time of sampling after the killing of the animal, ambient temperature, and time elapsing until delivery to the laboratory. Samples should be accompanied by information on the location where the sample has been taken and sex and age of the animal (Anonymous, 2009).

In disease free times serological surveys are an inexpensive and convenient tool to detect a fresh CSF outbreak. Specific antibodies persist lifelong in convalescent animals and only a limited number of samples is necessary to detect e.g., an expected prevalence of 5% with a 95% confidence. In contrast virological surveys would require a much higher number of samples to detect a similar low level of prevalence (Artois et al., 2002).

During a control program progress should be monitored very closely by investigating virologically and serologically the highest number of killed animals possible. Results of laboratory tests will yield an accurate account of control progress.

### Minimal intervention

In small isolated wild boar populations CSF outbreaks can be self-limiting as was observed in an outbreak in the southern part of Switzerland (Canton of Ticino). When the outbreak was notified the infected area was declared a “risk zone” and the surrounding area the “surveillance zone.” All hunting activities in the risk zone were temporarily banned, but hunting continued in the surveillance zone. Hunting was again allowed in the risk zone after 7 months and juvenile pigs were the major targets. Diagnostic and biological data of all 1294 wild boar found dead or shot between May 1998 and January 2000 were recorded and analyzed. Of 528 animals from the risk zone 179 were virus-positive and 167 seropositive, whereas only one animal was seropositive from the surveillance zone. After another year no more virus-positive animals were found. Seropositive animals were found in all age groups during the first hunting campaign. However, after 12 more months seropositive animals were all older than 1 year (Schnyder et al., 2002). Similar observations were made in neighboring Italian territories with a similarly structured wild boar population (Zanardi et al., 2003).

### Feeding

Feeding of wild boar is primarily used to facilitate trapping, shooting, or distraction of wild boar from crops (Massei et al., 2011). However, the latter is sometimes difficult or impossible, since additional feed is only accepted when feed in the natural environment is scarce. In order to achieve optimal results experienced hunters should decide on density and location of feeding stations. The common practice of artificial feeding in winter is not unambiguous, since it may contribute to survival and improved reproduction and thereby to population growth (Geisser and Reyer, 2005; Gamelon et al., 2011).

The usefulness of feeding for the control of CSF is also somewhat controversial, because—depending on the circumstances—it may promote or hamper the spread of CSFV. Feeding prevents local wild boar including infected animals from migrating to distant food sources thereby restricting the long distance spread of CSFV. On the other hand excessive artificial feeding could be an incentive for neighboring uninfected wild boar to move into the infected area. This may result in the spread of the virus into susceptible animals from formerly uninfected areas. However, cautious and limited use of feeding can be a useful tool to keep infected animals in a defined area and to limit spread of the virus (Artois et al., 2001).

### Fencing

Wild boar-proof fences have been described and they are being used on a small scale mainly to protect crops or certain ecological environments. Fencing could be also a method to effectively restrict wild boar movement in larger areas and thereby prevent the spread of CSFV. The efficiency of fencing is depending on the fencing system used and its intactness, since long fences of dozens of kilometers are vulnerable to destruction by wildlife and other influences. In addition the practical feasibility and public acceptance of implementing fencing in emergency situations in larger areas is limited. In case fencing is considered as an element of a control program, suitable areas for fencing must be identified taking into consideration the epidemiological situation and the spatial distribution of wild boar populations (Anonymous, 2014).

### Hunting

Several programs for controlling infectious diseases of wildlife have attempted the reduction of host population size in order to lower the density of both infected and susceptible individuals in a population. The goal was to achieve a low probability of transmission of infection between animals and to reach the specific threshold density of susceptible animals. Population reduction has been used in programs to control bovine tuberculosis in badgers in the United Kingdom, fox rabies throughout mainland Europe and CSF in wild boar in France, Germany, and Italy. In all cases population control was attempted by culling using different methods, e.g., hunting, gassing (foxes and badgers), trapping, and poisoning. In Europe there would be little or no public acceptance for the latter method since it is considered inhumane and non-target species may be affected. In addition there are no toxicants registered for this purpose in Europe or North America. For a number of years poisoning of wild boar was successfully applied in New Zealand and Australia (Massei et al., 2011). Another theoretical method to mitigate wild boar populations would be fertility control by feeding contraceptives in baits. However, the lack of long-acting contraceptives, possible uptake of baits by non-target species and the fact that wild boar meat is for human consumption prevented the use of this method. Modern immunocontraceptives are also unsuitable, since they have to be administered parenterally, i.e., using a remote delivery system for injecting each individual pig. A drawback of most campaigns for reduction of host animal populations is the lack of control of the target level of animal population decrease. Results of these campaigns so far were not sustainable (Aubert, 1999; Donnelly et al., 2003; Massei et al., 2011), partly because attempts to reduce population size by culling were often compensated by a higher reproduction activity and immigration from neighboring populations. If depopulation attempts do not reach the threshold density the infection will persist, probably at a lower level.

At first sight hunting seemed to be the method of choice for the reduction of the number of susceptible wild boar. However, in most countries wild boar are a major target species for sports hunting, which tends to maintain pig populations close to 50% of the level of the carrying capacity, thereby maximizing the production of new-born animals. This partly explains that—despite steadily increasing hunting bags all over Europe—wild boar populations have not decreased; in contrast they have increased over the last decades (Keuling et al., 2013). There is an obvious conflict between the goals of hunters and the goals of CSF control, which might impede control efforts considerably. Another problem is hunters' tradition not to shoot piglets or to hunt using artificial light. However, an effective hunting plan as part of a CSF control program must involve the preservation of adult animals that can be considered to be immune and the shooting of juvenile animals which are most likely susceptible to the virus. Targeting of piglets and young female pigs will have the most noticeable effect on the wild boar population (Bieber and Ruf, 2005; Toigo et al., 2007).

Considering the fast replication of wild boar the theoretically necessary reduction of >70–80% of the population could only be achieved through professional hunting campaigns with very high killing rates. In practice however, these rates are rarely if ever reached and in addition it must be considered that breeding rates following such a drastic reduction of population will be very high (Bieber and Ruf, 2005; Gamelon et al., 2011).

Therefore, hunting measures alone are not considered to be efficient for CSF control, but despite these limitations hunting can be useful as a complementary control measure (Zanardi et al., 2003) and necessary for collecting samples for laboratory diagnosis (Anonymous, 2009).

### Trapping

In smaller areas trapping can be used as an additional method for the reduction of susceptible wild boar. Different types of traps have been developed, which can be used to trap single wild boar or larger groups of pigs (Massei et al., 2011). The efficiency of the method was recently demonstrated in a forest area of 25 km^2^ in Bulgaria where a CSF outbreak had occurred. The density of wild boar was estimated to be 6 pigs/km^2^. Of a total of 156 animals 119 were removed by trapping within 3 months (Alexandrov et al., 2011). As a result the CSF infection chain was interrupted and the area became CSFV-free. The advantages of trapping are that it can be used in defined areas including residential areas, and (young) age classes can be removed selectively. The social disturbance in the population will be low compared to hunting. The disadvantages are that trapping is labor-intensive and it only works, when naturally occurring feed is scarce. Since it requires euthanasia, there might be little public acceptance and traps might be tampered with by adversaries.

Hunting and trapping not only leads to increased reproduction but it also might influence wild boar behavior with respect to an increase of their home range size and increased nightly activity (Scillitani et al., 2010).

### Oral immunization

Oral immunization of wild life species has proven to be very efficacious in the control of rabies. MLV or recombinant live vaccines were being used for that purpose. Oral vaccination of wild boar against CSF can also be used as a method to decrease the number of susceptible animals in an infected population.

**Figure 1:**
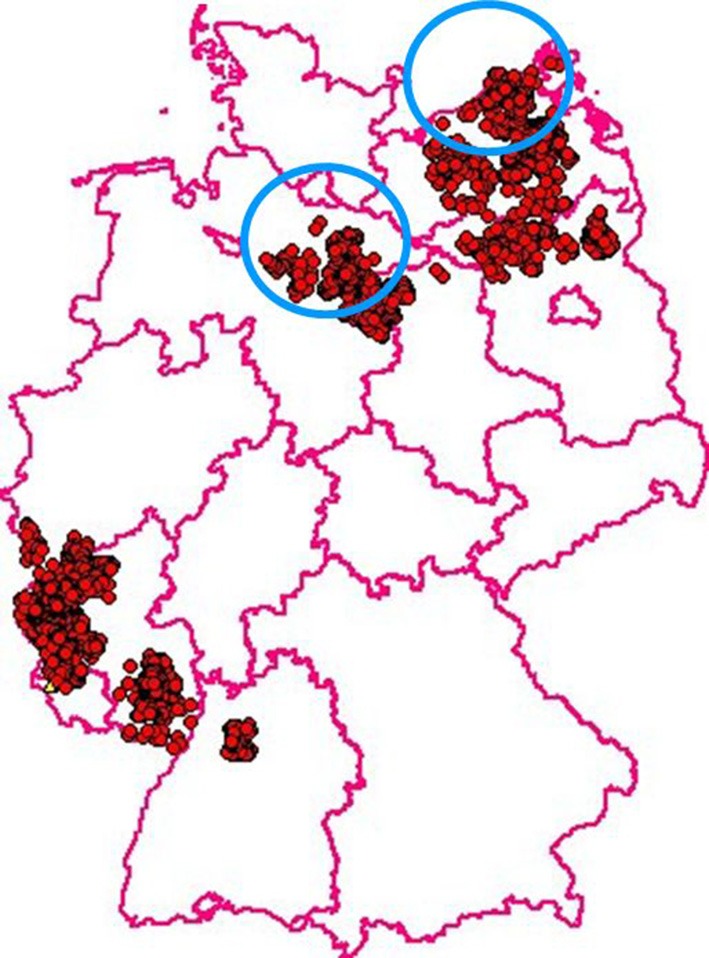
**CSF in wild boar in Germany: Between 1994 and 2008 3049 virus positive cases were recorded**. Blue circles indicate the regions where the two primary outbreaks were located in 1992. (Courtesy of Friedrich-Loeffler-Institute, Animal Disease Information System, TSN, 2015).

**Figure 2:**
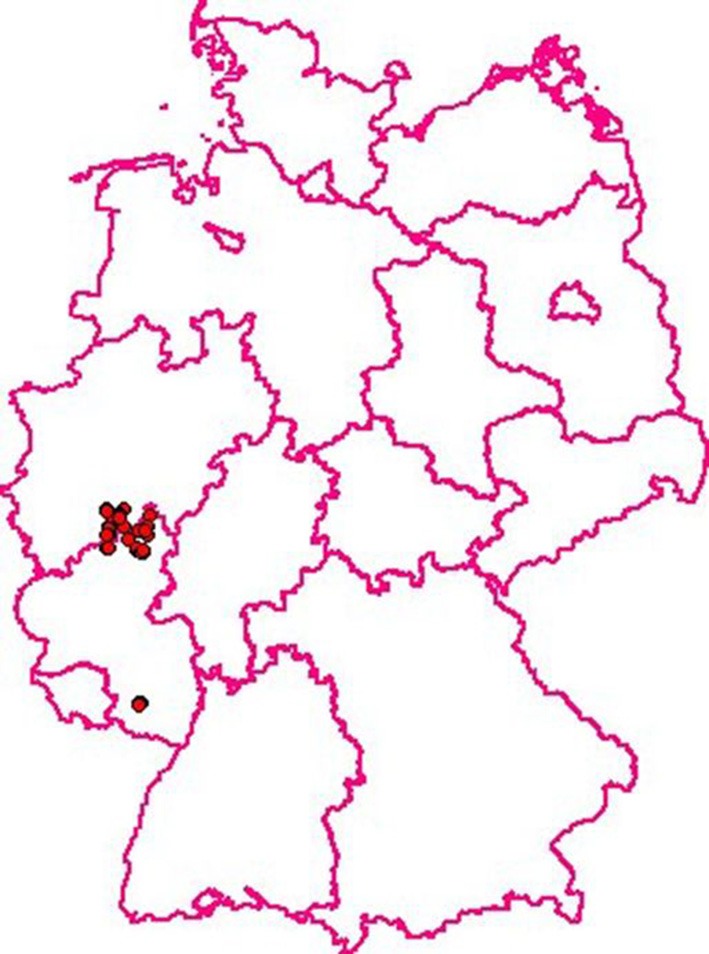
**CSF in wild boar in Germany: 52 cases were recorded between January and July 2009**. The last case was detected July, 29th 2009. Oral immunization was continued until spring of 2012. (Courtesy of Friedrich-Loeffler-Institute, Animal Disease Information System, TSN, 2015).

In 1992 two different variants of CSFV were introduced into the wild boar population by primary outbreaks in Northern Germany. The affected populations were dense and like the rest of the country they had been free from CSF. Conventional control measures, i.e., increased hunting pressure, hygienic measures, and the establishment of risk and surveillance zones failed to yield sustainable results and the infection spread and became endemic and—most likely by human interference—spilled over into more distant areas of the country. Between 1994 and 2008 a total of 3049 CSF cases were recorded in Germany (Figures 1, 2). In this critical situation the option of oral immunization was revisited and first experimental vaccination campaigns were started in 1994. After a few years the outcome of these experiments resulted in a protocol that can be summarized as follows:

The decision to use oral vaccination in the control of CSF should be made on the following criteria: (a) high infectious pressure when there are holdings of domestic pigs in the area; (b) CSF is endemic; (c) high density of wild boar and a high probability that a fresh CSF outbreak becomes endemic; (d) CSF outbreaks in natural reserves with restricted hunting.

The mode of bait distribution differs from oral vaccination of foxes and has to take into account the feeding characteristics of wild boar. Depending on the population structure 0.5–1 feeding place of about 200 m^2^ per 100 ha of hunting area is established. Wild boar should be attracted to the feeding place by laying out corn approximately 10 days prior to the distribution of baits. Thirty to forty baits are laid out by hand and covered with soil. Two to four weeks later an identical second vaccination is performed. Aerial distribution of baits is possible and can be applied in dense forests or difficult terrains where manual distribution would be difficult. After each vaccination hunting should be temporarily banned for at least 5 days and after the second vaccination feeding places should be inspected for residual baits in order to assess uptake rates. Usually, bait uptake is high and varies between 80 and 90%. Only when feeding places are not well accepted by the animals uptake rates can be lower (Kaden et al., 2002). The efficacy of oral vaccination should be assessed using serological monitoring. An extended ban of hunting in the vaccinated areas could help to maintain a stable population of predominantly immune animals for as long as possible. In case limited hunting activities are allowed they should concentrate on the shooting of piglets, because they cannot be immunized by oral immunization and they are the reservoir for the virus.

Initially two campaigns with two vaccinations each (double-vaccination) were used per year, i.e., in spring and autumn. However, the disadvantage was that only 20–30% of the young age class turned seropositive. Due to the natural behavior of wild boar adult animals are the first to pick up baits at the expense of the most important target animals, i.e., juvenile adults and weaned piglets. Apart from the competition with adult pigs these age groups can only be reached from an age of 4 months and older depending on bait size. Consequently frequent campaigns are necessary and only after introduction of a third double-vaccination campaign in summer piglets born in spring can be reached at the age of 5 months and more than 50% of young animals shot at the age of approximately 6 months can be found seropositive.

When deciding where to vaccinate there are several possible scenarios: Vaccination in the infected area is applied in order to increase population immunity in order to reach the threshold of *R*_0_ < 1. In addition the surrounding surveillance area may be vaccinated simultaneously in order to prevent virus spread outside the infected area. Alternatively, only the surrounding area may be vaccinated as a prophylactic measure in order to stop spread of the virus outside the infected area (*cordon sanitaire*). In the latter case no particular measures are taken in the infected area when there is reason to believe that the infection will fade out over time without intervention (Kaden and Lange, 2001).

Assuming that the threshold for *R*_0_ < 1 is reached at a level of about 200 seronegative pigs in an area of approximately 220 km^2^, this value can be reached when 500 wild boar living in that area show a seroconversion of 60%. With higher numbers of wild boar seroconversion rates must increase accordingly (Artois et al., 2002).

In Germany results of oral immunization of wild boar varied considerably. In four federal states maximum seroconversion rates after three vaccination campaigns ranged between 37 and 72%. A distinction between antibodies against field virus and vaccine virus was not possible, but it was clearly shown that seroconversion levels rose after each oral vaccination campaign. Virus prevalence was highest in pigs < 1 year (79–88%), in the age class 1–2 years prevalence varied between 9 and 19%, whereas adult animals >2 years were rarely found virus-positive (Kaden et al., 2002). Control programs should last at least 2 years and the vaccination area has to be large enough to include animal movements (Kaden et al., 2002). After introduction of oral vaccination all outbreaks of endemic CSF in wild boar in Germany were eradicated within a few years.

A direct assessment of the efficacy of hunting measures alone vs. oral vaccination combined with hunting was carried out in the German federal state of Rhineland-Palatinate from 1999 to 2005. For 3 years after notification of the CSF outbreak in wild boar the control was based on increased hunting, in particular juvenile pigs. General hygiene measures were part of the control plan. Both measures had no noticeable effect on the endemic persistence of CSF. From 2002 until the end of the study the strategy was changed and oral immunization was started as a new major control tool (von Rüden et al., 2008). In parallel wild boar found dead and shot pigs in the restriction area, totaling over 110,000 animals, were tested virologically and serologically for CSF. The laboratory records contained information about geographical origin, gender, and age of the pigs. About 82% of all virologically positive animals were piglets, thus clearly demonstrating that these animals were the virus reservoir and responsible for perpetuating the epidemic/endemic. When the hunting bag was analyzed it became clear that during the whole control program older animals were overrepresented and that not enough young pigs had been shot. This was a clear proof that despite all awareness programs local hunters did not fully support the control program. In piglets the virological prevalence was higher and the serological prevalence was lower compared to adult pigs and yearlings before the start of oral immunization. These differences were significant. After the start of the oral immunization campaign in February 2002 virus prevalence decreased markedly and seroprevalence increased considerably all over the age classes. The last virus-positive wild boar was recorded in July 2009 (von Rüden et al., 2008). In retrospect it is safe to assume that the introduction of oral immunization of wild boar against CSF was a most crucial factor for the eradication of the infection from the German wild boar population (Figures 1, 2).

Criteria for the lifting of restrictions are the last virologically positive case and the serological status of juvenile animals. When all young wild boar, after waning of maternal immunity, are seronegative and the last virus-positive animal was found more than a year ago it can be assumed that the infection has faded out.

## Outlook

The control of CSF in wild boar has significantly improved during the last three decades, and a number of tools for the control of CSF are available and strategies have been developed to eradicate the infection in dense wild boar populations. However, there are several details worth amending: In order to enhance sensitivity of virus isolation from organ samples from wild boar, the use of RNA transfection could be introduced routinely (Meyer et al., 2015). This might also minimize bacterial contamination problems often associated with field samples from hunters.

Vaccination plays a major role in the inventory of control measures (Rossi et al., 2010). Two major problems are still associated with oral vaccination: Due to the hierarchical structure of wild boar families old animals tend to eat most baits at feeding places. This could only be solved by devising mechanical barriers that can only be bypassed by small piglets. In addition bait size hinders the acceptance by piglets. Smaller baits might lower the age of pigs that can be reached with oral vaccination. Since present vaccines are based on conventional MLVs there is no distinction possible between infected and vaccinated animals. For future vaccination campaigns it would be desirable to have a DIVA vaccine for oral vaccination available. The serological distinction of vaccinated pigs would greatly facilitate monitoring of progress of control programs.

### Conflict of interest statement

The author declares that the research was conducted in the absence of any commercial or financial relationships that could be construed as a potential conflict of interest.
